# MIND the GAP: Psychiatry's Scottish Trainee Enhanced Programme (STEP) to Reduce Differential Attainment

**DOI:** 10.1192/bjo.2024.286

**Published:** 2024-08-01

**Authors:** Michael Cooper, Rekha Hegde, Fahd Cheema

**Affiliations:** 1 NHS Lanarkshire, East Kilbride, United Kingdom; 2 NHS Greater Glasgow and Clyde, Glasgow, United Kingdom

## Abstract

**Aims:**

Build relationship and understanding between International Medical Graduates (IMGs) and supervisors in core psychiatry through the Scottish Trainee Enhanced Programme (STEP) to help facilitate belonging and support a vulnerable group. Thus improving training outcomes.

**Methods:**

There are various different areas to the PsychStep Programme.

A handbook is distributed to all IMGs which has been created in accordance with the Royal College of Psychiatrists (RCPsych) IMG Guide.

A trainee created video guide for the RCPsych portfolio is provided with the aim of this reducing anxiety around its use.

A Scotland wide WhatsApp group was created for peer support.

The final part of the programme was attendance of trainees and crucially supervisors at two half day sessions. Joint attendance is crucial in fostering supportive relationships. Content was delivered via workshops by IMG consultants with lived experience. Topics covered included communication skills, success factors, reflection, cultural transition and cultural competence. These sessions were evaluated using both scale questions and free text.

**Results:**

There were 6 participants in this programme. All participants reported on evaluation that they felt this course made them:

- Feel welcome to psychiatry.
- Realise that other trainees faced the same challenges as them.
- Felt supported in their Journey.

All participants stated that their trainer attended the virtual sessions with them and that them being in attendance helped them understand specific challenges they would face as an IMG.

Themes identified on free text feedback from trainees were advice on how to reflect and the support that is available in general. The opportunity for shared personal experiences was also highlighted as a positive.

Themes identified on trainer feedback were guidance around both provision of a supportive environment and the importance of having open discussions with trainees.

**Conclusion:**

This programme has an important role in helping IMG trainees in psychiatry succeed. Success stories of IMG consultants provided knowledge and hope for them. The IMG trainees felt better able to effectively engage with supervision following participation in this programme.

Outcomes from this programme to date highlight opportunities to build on this in the future. The organisers of this course hope to increase future attendance and share good practice across other specialities.

